# Screening and Functional Analysis of *TPO* Gene Mutations in a Cohort of Chinese Patients With Congenital Hypothyroidism

**DOI:** 10.3389/fendo.2021.774941

**Published:** 2021-12-21

**Authors:** Huijjuan Wang, Wenxia Wang, Xi Chen, Hailong Shi, Yinmin Shi, Guifeng Ding

**Affiliations:** ^1^ The National Engineering Research Center for Miniaturized Detection Systems, Northwest University, Xi’an, China; ^2^ Pediatrics, Urumqi Maternal and Child Health Care Hospital, Urumqi, China; ^3^ College of Basic Medicine, Shaanxi University of Chinese Medicine, Xianyang, China; ^4^ Obstetrics and Gynecology Department, Urumqi Maternal and Child Health Care Hospital, Urumqi, China

**Keywords:** congenital hypothyroidism (CH), thyroid peroxidase (TPO), gene mutation, genotype, phenotype

## Abstract

**Backgrounds:**

As a crucial enzyme in thyroid hormone synthesis, the genetic defective thyroid peroxidase (*TPO*) was one of the main genetic factors leading to congenital hypothyroidism (CH).

**Methods:**

Mutations in the *TPO* gene were screened and identified in 219 patients with CH from northwest China by using high-throughput sequencing and bioinformatics analysis. The biological function of detected variants was studied by *in vitro* experiments and homology modeling.

**Results:**

Nineteen rare variants, including seven novel ones, were detected in 17 of 219 patients (7.8%). Most cases were detected with one single heterozygous variant, and only two patients were detected with multiple variants, i.e., compounds for (1) IVS7-1G>A, p.Ala443Val, and p.Arg769Trp and (2) p.Asn592Ser and p.Asn798Lys. The biological function of the four missense mutations (i.e., p.Ala443Val, p.Arg769Trp, p.Asn592Ser, and p.Asn798Lys) they carried were further studied. Experimental data showed that these four mutations did not affect the protein expression level of the *TPO* gene but remarkably reduced the peroxidase activity toward guaiacol oxidation, retaining 8–32% of activity of the wild-type protein. The comparison of the predicted 3-D structures of wild-type and mutant TPO proteins showed that these four amino acid substitutions changed the non-covalent interactions of studied residues that might alter the structure and function of the TPO protein.

**Conclusion:**

This study was the first to analyze the *TPO* mutation spectrum of patients with CH in northwest China. Our data indicated that the *TPO* mutation was not a common reason to cause CH in China. The functional data may help to clarify the structure-function relationship of the TPO protein and provide further evidence for the elucidation of the genetic etiology of CH.

## Introduction

Congenital hypothyroidism (CH) is one of the most frequent neonatal endocrine and metabolic diseases and affects approximately 1:2,000–1:4,000 infants worldwide (1). As a preventable complex disease, highly variable clinical manifestations and symptoms are observed among different individuals (1, 2). Although the precise pathogenic mechanism of CH is not elucidated, a genetic origin in the pathogenesis has been widely acknowledged, and a proportion of patients have hereditary diseases caused by genetic abnormalities (3–5). To date, a number of genes associated with CH have been identified (6, 7). Genetic defective thyroid peroxidase (*TPO*) is considered as one of the most common causes of thyroid dyshormonogenesis (1, 4).

TPO is a thyroid-specific heme peroxidase localized in the apical membrane of thyrocytes that catalyzes the oxidation and organization of iodide in the tyrosine residues of thyroglobulin and forms active thyroid hormones by the coupling of iodotyrosine residues (8, 9). Considering the key role of TPO in thyroid hormone biosynthesis, mutations in the *TPO* gene can impair thyroid hormone production due to total (TIOD) or partial (PIOD) iodide organification defects (9, 10). Cases of CH due to *TPO* genetic defects are usually inherited in an autosomal recessive manner and are often manifested as permanent CH and goiter (9–11). Severe defective *TPO* bioallelic mutations were considered to lead to TIOD, and PIOD is generally related to the heterozygous *TPO* mutation (9, 12). However, single *TPO* mutations were detected in some patients with TIOD, and multiple *TPO* mutations were detected in some patients with PIOD, indicating a lack of direct correlation between *TPO* genotypes and phenotypes (9, 12–14). In addition, the frequency and distribution of *TPO* mutations in patients with CH seem to be specific to ethnicity. In patients with thyroid dyshormonogenesis, the detection rate of *TPO* mutations is 20–46% in Caucasians (15–17) but only less than 10% in East Asians (18–23). To date, more than 170 mutations in the *TPO* gene have been recorded in the Human Gene Mutation Database (HGMD professional, 2021.2). However, the biological function of most of the identified *TPO* mutations remains unknown. Therefore, comprehensive studies covering different populations and in-depth functional studies are helpful to have a thorough understanding of the role of *TPO* mutation in the pathogenesis of CH.

According to the newborn screening data, the incidence rate of CH in China is higher than the global average level (24, 25) and is even higher in northwest China. A rate of 1 in 1,468 newborns was reported in Xinjiang (26), which accounts for one-sixth of China’s territory. Currently, the *TPO* mutation spectrum in patients with CH of these regions is not studied yet. Therefore, in this study, *TPO* mutations in 219 patients with CH from northwest China are comprehensively screened using high-throughput sequencing to obtain the *TPO* mutation spectrum of patients with CH in northwest China and explore the genotype–phenotype relationship. Moreover, the function of some important mutations is investigated through a series of *in vitro* experiments and molecular modeling. The findings obtained in this study will facilitate the genetic counseling and prognosis of patients with CH and will be helpful for clarifying the molecular mechanism underlying CH pathogenesis caused by *TPO* mutation(s).

## Materials and Methods

### Study Samples

A total of 219 patients with CH were recruited in Urumqi Maternal and Child Health Care Hospital, Xinjiang, China, from October 2017 to June 2019. The inclusion criteria were as follows: positive neonatal screening with a diagnosis of CH confirmed by serum thyroid function tests at 2–4 weeks of age (27). In brief, all patients undergoing neonatal screening with TSH levels of >10 uIU/ml, elevated serum TSH (>7.5 uIU/ml), and decreased FT4 levels (0.5–7.1 pmol/L) at diagnosis were finally included. Thyroid ultrasonography and 99mTc scintigraphy were performed during the neonatal period prior to treatment. Patients with thyroids width ≥+2SD (standard error) in size were defined as goiters, with thyroids ≤−2SD were considered as hypoplasia. The parents of all participants gave written informed consent in accordance with the Declaration of Helsinki. The study was approved by the Medical Ethics Committees of Urumqi Maternal and Child Health Care Hospital.

### DNA Extraction and Sequencing

Genomic DNA was extracted from the peripheral blood samples collected from recruited patients. Recruited patients were genetically screened with a customized Ampliseq panel that included 29 CH-associated genes as described previously (27). The coding exons and the 20 flanking base pairs of splice junctions surrounding the exons of the targeted genes were sequenced using the Ion Torrent PGM instrument (Thermo Fisher Scientific, MA, USA). For the *TPO* gene, 21 amplified amplicons were obtained at each sequencing run. The amplicon length ranged from 166 bp to 374 bp.

### Variant Detection and Interpretation

Raw data were processed using the Torrent Suite software (version 5.0.4; Life Technologies), and the Ion Reporter (https://ionreporter.lifetechnologies.com/ir/secure/home.html) and ANNOVAR package (http://wannovar.wglab.org/) were used for variant annotation. Variants were filtered in accordance with the criteria described in our published study (27). In brief, non-synonymous variants with minor allele frequencies (MAFs) of ≤0.01 or no MAF values in the public databases, such as dbSNP database (http://www.ncbi.nlm.nih.gov/projects/SNP/), 1000 Genomes Project (http://ftp.ncbi.nih.gov/), Exome Sequencing Project (http://evs.gs.washington.edu/EVS/), and the Genome Aggregation Database (http://gnomad.broadinstitute.org/), were selected. All selected variants were validated through the Sanger sequencing with the ABI3500 xL Dx (Applied Biosystems, Foster City, CA, USA).

The effects of detected variants were assessed using *in silico* programs. For missense or indel variants, four *in silico* tools, including Polymorphism Phenotyping v2 (http://genetics.bwh.harvard.edu/pph2/index.shtml), Mutation Taster (http://www.mutationtaster.org/), SIFT (http://provean.jcvi.org/genome_submit_2.php), and Mendelian Clinically Applicable Pathogenicity (http://bejerano.stanford.edu/MCAP/), were used. The pathogenicity of each variant was assessed in accordance with the standards described by the American College of Medical Genetics (ACMG) (28).

The protein sequences of TPO from various species and other members of the peroxidase superfamily were downloaded from NCBI (https://www.ncbi.nlm.nih.gov/protein/). Alignments were performed using the DNAMAN version 9 (Lynnon Biosoft, Quebec, Canada). The protein domain and structure of TPO were obtained from the UniProt Knowledgebase (https://www.uniprot.org/).

### Homology Modeling of Human TPO Protein

A three-dimensional homology model of human TPO was constructed using the Modeller 9.13 software (https://salilab.org/modeller/). The crystal structure of Human Myeloperoxidase (PDB id: 6BMT) was used as template for TPO homology modeling because of the highest amino acid homology (42% sequence similarity). The same software was used to generate a homology model of the human TPO containing the detected mutation for structural comparison. Both models were then subjected to molecular dynamics simulation by using the Gromacs 2019 software (https://manual.gromacs.org/) with CHARMM27 all-atom force field and explicit water environment neutralized by counter ions at 310 K. Structures with the lowest potential energy were extracted and verified using Ramachandran plots, which were generated using the Procheck software (http://nihserver.mbi.ucla.edu/SAVES), to check the stereochemical quality of the protein. The Verify3D software was used to determine the compatibility of the 3D atomic model with its own 1D amino acid sequence, and the Errat software (http://nihserver.mbi.ucla.edu/SAVES) was used to analyze the statistics of non-bonded interactions among different atom types. The BIOVIA Discovery Studio Visualizer (www.3ds.com, Dassault Systèmes; San Diego, CA, USA) was used for viewing, sharing, and analyzing protein data.

### *In Vitro* Functional Analysis of TPO Mutants

Th pEGFP-C1-based vectors containing *TPO* genes with different genotypes, including wild-type (wt) and five mutant *TPO* (i.e., p.Glu337Lys, p.Ala443Val, p.Asn592Ser, p.Arg769Trp, and p.Asn798Lys), were constructed. HEK 293T cells were maintained in Dulbecco’s Modified Eagle Medium (Gibco, Life Technologies) supplemented with 50 U/ml penicillin–streptomycin, 2 mM L-glutamine, and 10% fetal bovine serum (Gibco, LifeTechnologies). Cells were seeded in a 24-well plate (1 × 10^5^ cells per well) and transfected with plasmids by using the Lipofectamine 2000 (Invitrogen). Cells were harvested after 24 h transfection, washed twice with phosphate buffered saline (PBS), and lysed using a sonicator in PBS (pH = 7.4) in ice. Sonicated extracts were subjected to centrifugation for 5 min at 1,000 g (4°C). The supernatant was transferred into a new tube and quantified through the Bradford assay (HyClone-Pierce, USA) by using bovine serum albumin as a standard in accordance with the manufacturer’s protocol.

For immunoblot analysis, 60 μg supernatant prepared from the cells transfected for 24 h were separated by 10% SDS-PAGE. Western blotting was performed with an anti-His tag rabbit monoclonal antibody (Cell Signaling Technology, MA, USA) used at 1:1,000 dilution. Horseradish peroxidase-labeled goat antirabbit antibody IgG (1:5,000 dilution; ABclonal, China) was used as a second antibody. Immunoreactive proteins were visualized using the SuperSignal West Femto Trial Kit (Thermo Fisher Scientific Inc., Rockford, IL, USA), and band densities were determined with the BIO-1D software (SIM International Group Co., Newark, NJ, USA). The relative protein level of each detected sample was corrected to the β-actin expression and expressed in relation to wt TPO protein (i.e., relative protein level of wt TPO was 100%). All assays were performed in triplicate with independent preparations.

The guaiacol oxidation assay was performed to measure the enzymatic activity of wt and mutant TPO proteins (29). The reaction mixture contained 10 μl supernatant, 5 mmol/L guaiacol, and 892 mM H_2_O_2_ in PBS. The reaction was initiated with the addition of H_2_O_2_, and absorbance was monitored spectrophotometrically at 470 nm every 60 s for more than 30 min. The reaction velocity was estimated from the slope of the initial linear part (*d*A*/d*t) of the curves obtained, and the specific enzyme activity of each tested sample was expressed as *d*A·min^−1^·mg^−1^. Data were representative of at least three independent experiments (each performed in triplicate) with similar results.

### Statistical Analysis

Statistical analysis was performed by using the GraphPad Prism software (version 5.01; GraphPad Software, Inc., San Diego, CA, USA). The one-way analysis of variance with Dunnett’s test was used to evaluate the differences in the enzymatic activity between wt and mutant TPO enzymes. P < 0.05 was considered statistically significant.

## Results

### *TPO* Mutation Spectrum Analysis

Through the genetic screening of 219 patients with CH, 19 rare variants (with MAF <1% in public population databases) in the *TPO* gene were found in 17 subjects with a mutation detection rate of 7.76% (17/219). These variants included 17 missense mutations, 1 non-sense mutation, and 1 splicing variant. Of these variants, 12 were reported in the published literature and databases. Six of these variants were identified in the reported cases of published studies and considered as (likely) pathogenic mutations, and the six other variants were reported in the public population databases but not reported in patients. Seven variants, including 1 splicing variant (i.e., IVS7-1G>A) and 6 missense mutations (i.e., p.Arg279Trp, p.Ser309Pro, p.Ala443Val, p.Ser571Arg, p.Asn798Lys, and p.Ser853Leu; **Tables 1** and **2**), were first found in this study.

**Table 1:** Clinical information of CH patients with *TPO* variants.

Patient ID	Date of birth	Birth weight (g)	Gestational age (week+day)	Gender	Thyroid	Neonatal screening		Diagnostic		Severity	TPO mutation
						TSH (uIU/ml)	Age	TSH (uIU/ml)	FT4 (pmol/L)		
E150012	2012/1/2	2,900	37	Female	Hypoplasia	35.4	69d	>100	1.20	Severe	p.Arg361Leu
E150018	2014/10/29	3,500	41	Male	Normal	>100	20d	>100	0.70	Severe	p.Glu757*
E150027	2014/4/15	3,500	40+6	Male	Goiter	19	61d	58.21	4.50	Moderate	p.Arg846Trp
E150041	2009/11/14	3,000	37	Male	Goiter	18	58d	20	6.40	Mild	p.Ser571Arg
E150048	2014/10/24	3,750	41+2	Male	Goiter	35.1	78d	35	4.90	Moderate	p.Pro883Ser
E160002	2014/11/8	NA	NA	Female	Athyreosis	46.1	64d	>100	1.90	Severe	p.Pro883Ser
E160006	2015/5/16	1,900	38	Female	Normal	15	33d	15	7.10	Mild	p.Ser309Pro
E160053	2014/3/2	3,950	41+4	Male	Normal	223	19d	>100	3.73	Severe	p.Pro135His
E160078	2015/7/8	3,100	NA	Female	Goiter	44	24d	15.33	2.34	Severe	p.Asn674Ser
E160089	2016/6/20	3,300	42	Male	Normal	11.9	25d	13.4	8.80	Moderate	p.Gly889Arg
E160095	2015/7/5	3,800	40	Male	Goiter	>100	74d	>100	0.60	Moderate	p.Arg846Trp
E160098	2011/8/16	3,780	40	Female	Normal	646	28d	>100	1.29	Severe	IVS7-1G>A, p.Ala443Val, p.Arg769Trp
E160115	2016/8/27	2,900	39+3	Female	Normal	168	43d	25.89	6.95	Moderate	p.Arg279Trp
E160129	2016/8/30	4,100	37+4	Female	Goiter	275	57d	>100	1.42	Severe	p.Glu337Lys
E170009	2016/11/27	3,900	40	Female	Normal	74	43d	>100	4.76	Severe	p.Asn592Ser, p.Asn798Lys
E170026	2007/6/4	NA	NA	Female	Normal	10	20d	15.8	7.40	Moderate	p.Ser853Leu
E170076	2016/3/23	NA	NA	Male	Normal	15	22d	23	6.80	Moderate	p.Glu673Lys

NA, not available; p.Glu757*, *was used to describe a stop codon.

**Table 2:** Detailed information of detected *TPO* variants.

Physical position	Exon	rs ID	cDNA change	Amino acid change	Number of patients (n)	Frequency in public database		Status	ACMG pathogenicity classification
						gnomAD (East Asian)	1000 Genome (CHB)		
chr2:1544412	16	rs546683738	c.2665G>A	p.Gly889Arg	1	0.0003492	0	Known^a^	VUS
chr2:1544394	16	rs190968346	c.2647C>T	p.Pro883Ser	2	0.005409	0.0146	Known^b^, DM	LP
chr2:1520694	15		c.2558C>T	p.Ser853Leu	1	0.0001157	/	Novel	LP
chr2:1520672	15	rs28913014	c.2536C>T	p.Arg846Trp	2	0.00159	0.0049	Known^a^	VUS
chr2:15003965	14		c.2394C>G	p.Asn798Lys		/	/	Novel	LP
chr2:1500456	13	rs114406277	c.2305C>T	p.Arg769Trp	1	0.003235	0	Known^a^	LP
chr2:1500418	13	rs770781635	c.2268dupT	p.Glu757*	1	0.00159	/	Known^b^, DM	P
chr2:1499775	12	rs780437569	c.2021A>G	p.Asn674Ser	1	0	/	Known^a^	VUS
chr2:1499771	12	rs201193196	c.2017G>A	p.Gly673Lys	1	/	0.0049	Known^b^, DM?	VUS
chr2:1497754	11	rs200122184	c.1949G>A	p.Gly650Glu	1	0.0001156	0.0049	Known^a^	VUS
chr2:1497580	11	rs755047215	c.1775A>G	p.Asn592Ser	1	/	/	Known^a^	LP
chr2:1491708	10		c.1713C>G	p.Ser571Arg	1	/	/	Novel	VUS
chr2:1481366	8		c.1328C>T	p.Ala443Val	1	/	/	Novel	LP
chr2:1481120	8	rs201781919	c.1082G>T	p.Arg361Leu	1	0.009273	0.0194	Known^b^, DM	LP
chr2:1477275	8		c.1009G>A	p.Glu337Lys	1	/	/	Known^b^, DM	P
chr2:1480963	8		c.925T>C	p.Ser309Pro	1	/	/	Novel	VUS
chr2:1477101	8		c.835C>T	p.Arg279Trp		/	/	Novel	VUS
chr2:1480857	Intron7		c.820-1G>A	IVS7-1G>A	1	0.00005722	/	Novel	P
chr2:1440078	4	rs61758083	c.404C>A	p.Pro135His	1	/	/	Known^b^, DM?	VUS

p.Glu757*, *was used to describe a stop codon.

a Variants were reported in public population databases, such as dbSNP, ExAC, or 1000 Genomes Project but without phenotypic data and pathological assessment.

b variants were reported in the published literature as well as HGMD (professional version 2021.2); DM, disease-causing mutation; DM?, a possible disease-causing mutation; P, pathogenic; LP, likely pathogenic; VUS, variants of uncertain significance.

**Figure 1:**
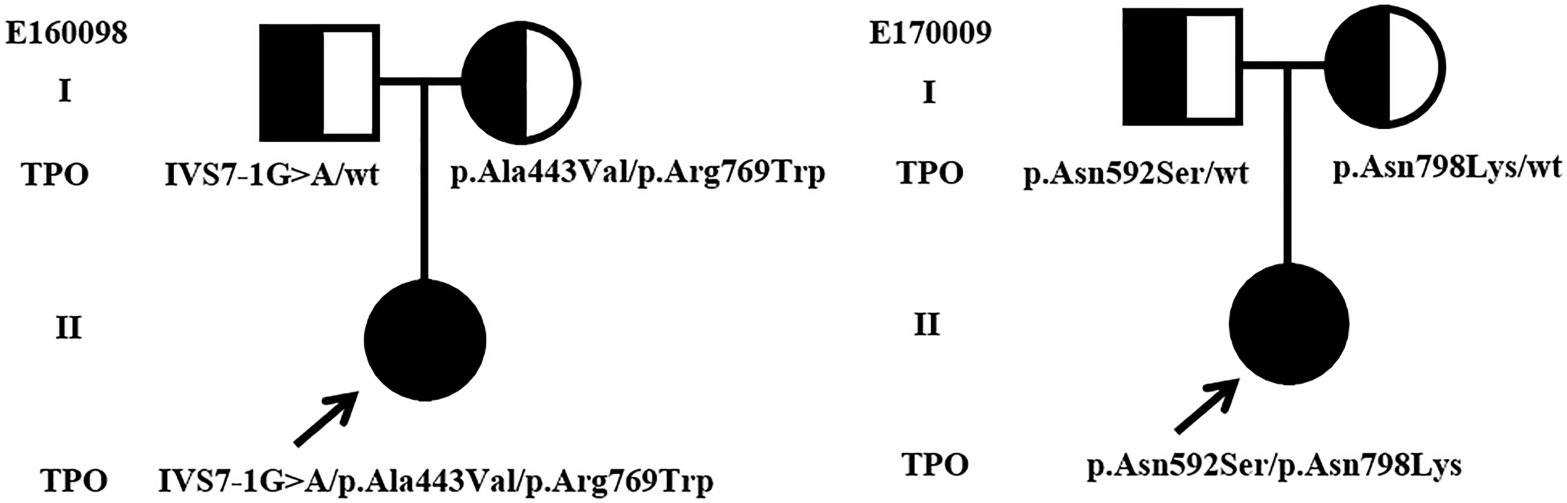
Genotypes and pedigrees of two CH patients. Arrow, Proband; Roman numbers, generations; squares, males; circles, females; half-filled symbols, unaffected heterozygote individuals.

Most of the detected variants were only found in one patient, whereas p.Pro883Ser and p.Arg846Trp were found in two cases. c.2268dupT, which was reported as a hotspot mutation in Taiwanese and Malaysian patients with CH (10, 30), was only found in one patient. Majority of patients carried one heterozygous variant, and only two cases (E160098 and E170009) carried multiple heterozygous variants (**Table 1**), i.e., compounds for (1) IVS7-1G>A, p.Ala443Val, and p.Arg769Trp and (2) p.Asn592Ser and p.Asn798Lys. The family pedigree analysis showed that of the variants harbored by E160098, IVS7-1G>A was inherited from her father, and p.Ala443Val and p.Arg769Trp were inherited from her mother. Of the variants carried by E170009, p.Asn592Ser was inherited from her father, and p.Asn798Lys was inherited from her mother (**Figure 1**).

Of the 17 cases with *TPO* variants, 9 presented normal thyroid shape and size, 6 were diagnosed with goiter, 1 showed thyroid dysplasia, and 1 was suspected with athyreosis. On the basis of serum FT4 levels at diagnosis, CH could be categorized as severe (FT4 < 5 pmol/L), moderate (5 pmol/L ≤ FT4 < 10 pmol/L), or mild (FT4 ≥ 10 pmol/L) (31). The two cases with multiple *TPO* variants were severe CH but with normal thyroid. Of the cases carrying one variant, 9 had moderate or mild CH, and 6 had severe CH. Two cases had the same mutation p.Pro883Ser, but their phenotype was different. The first case had moderate CH with goiter, and the other case had severe CH with athyreosis.

### Pathogenicity Evaluation of Detected Variants

On the basis of available evidence, the pathogenicity of detected variants was classified in accordance with the ACMG standards and guidelines. Of 19 detected variants, 10 variants were classified as pathogenic (P) or likely pathogenic (LP), including IVS7-1G>A, p.Glu757*, p.Pro883Ser, p.Ser853Leu, p.Asn798Lys, p.Arg769Trp, p.Asn592Ser, p.Ala443Val, p.Arg361Leu, and p.Glu337Lys. Other nine variants were classified as variant of uncertain significance (VUS). Among the seven novel variants, four (IVS7-1G>A, p.Ala443Val, p.Asn798Lys, and p.Ser853Leu) were evaluated as P or LP, and the other was classified as VUS (**Table 2** and **Supplementary Table 1**).

### *In Silico* Analysis of Effects of the Detected Variants

**Figure 2:**
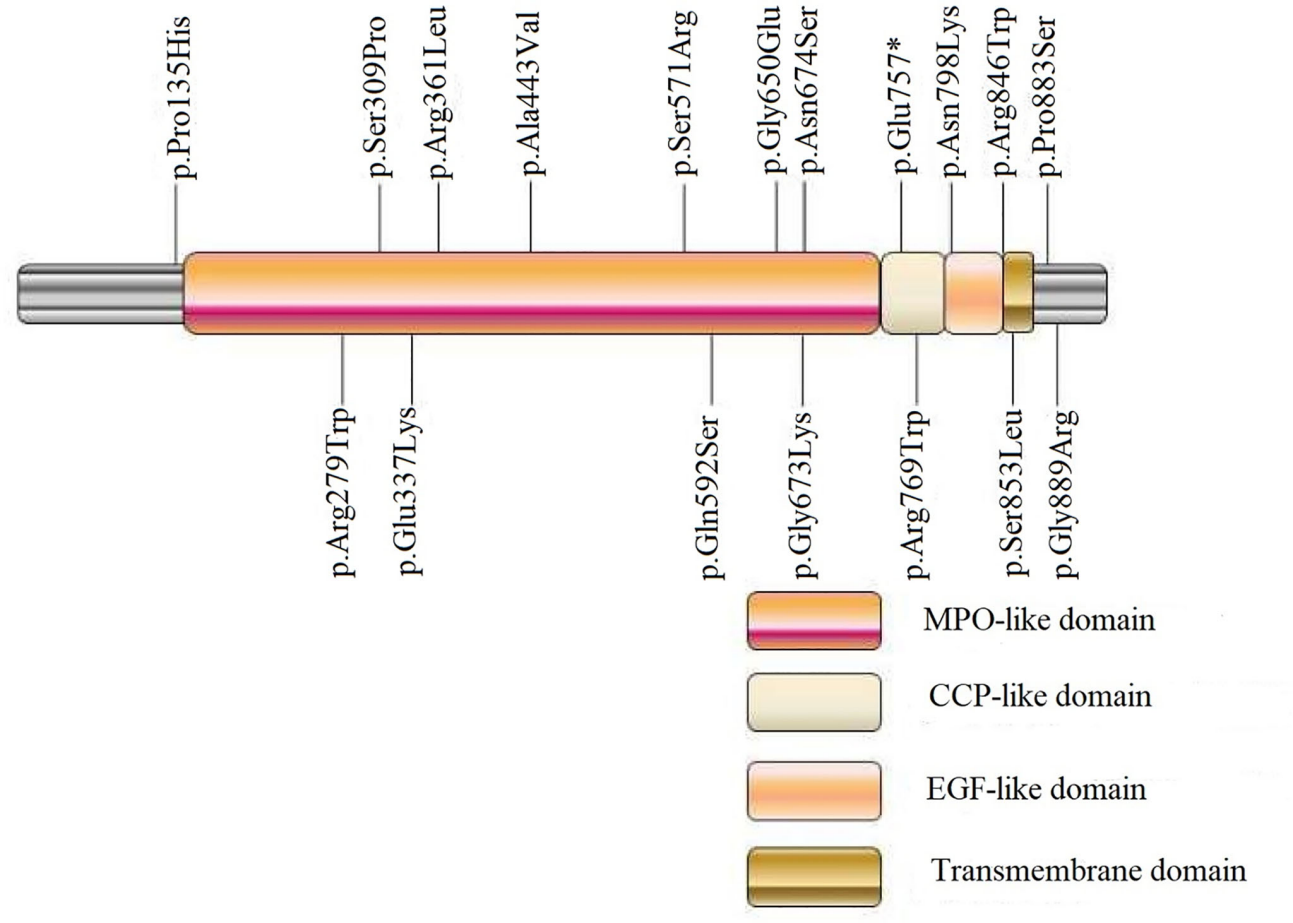
Schematic diagram of detected exonic variants in TPO protein domains. MPO, myeloperoxidase; CCP, complement control protein; EGF, epidermal growth factor. p.Glu757*, * was used to describe a stop codon.

Of the detected variants, p.Pro135His was located at the N-terminal of TPO protein, and p.Gly889Arg and Pro883Ser were located at the C-terminal tail (intracellular domain). p.Asn798Lys was located at the epidermal growth factor (EGF)-like domain; p.Arg769Trp and p.Glu757* were located at the complement control protein (CCP)-like domain; p.Asn674Ser, p.Gly673Lys, p.Gly650Glu, p.Asn592Ser, p.Ser571Arg, p.Ala443Val, p.Arg361Leu, p.Glu337Lys, p.Ser309Pro, p.Arg279Trp were located at the myeloperoxidase (MPO)-like domain (catalytic domain); and IVS7-1G>A was located in the intron near the catalytic active center (**Figure 2**).

The possible functional effects of detected missense mutations were assessed by *in silico* programs, including SIFT, PolyPhen-2, MutationTaster, and M-CAP. As shown in **Supplementary Table 2**, p.Gly673Lys, p.Gly650Glu, p.Asn592Ser, and p.Ala443Val were predicted by all software programs to have a deleterious effect on the TPO protein, whereas p.Pro883Ser was harmless to the protein function. The prediction results of other variants were inconsistent among different software programs.

**Figure 3:**
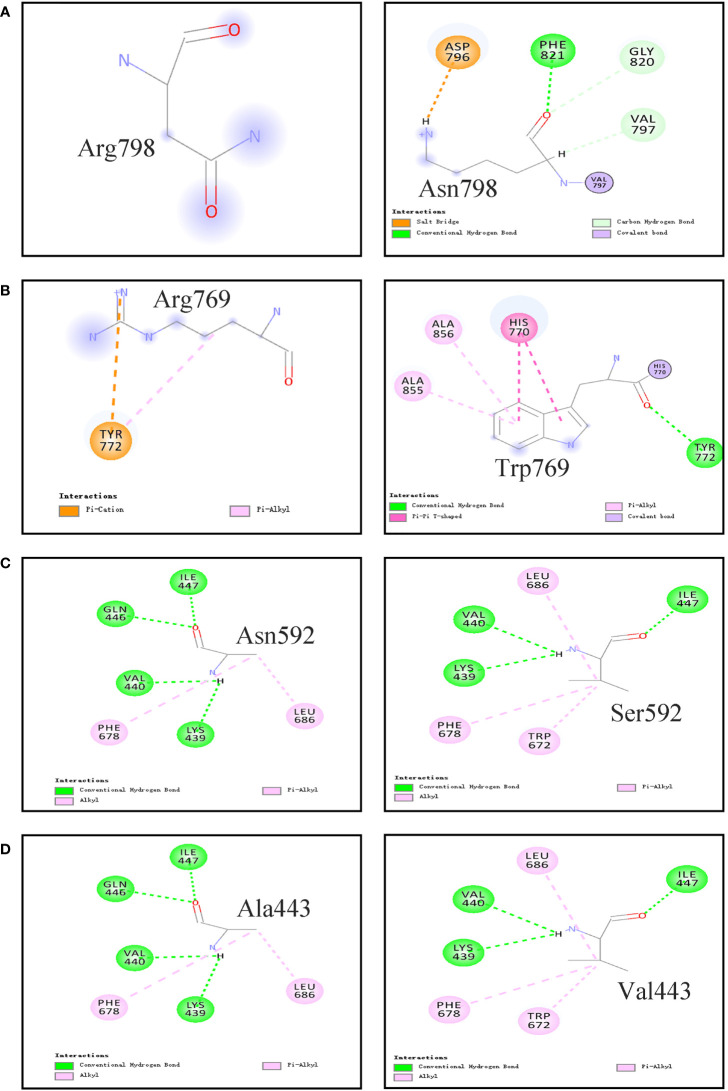
Non-covalent Interactions between the studied residues with surrounding residues in the TPO protein which are generated by computer. **(A)** p.Asn798Arg; **(B)** p.Arg769Trp; **(C)** p.Asn592Ser; **(D)** p.Ala443Val.

The 3D homology model of the wt TPO and four mutant (i.e., p.Asn798Lys, p.Arg769Trp, p.Asn592Ser, and p.Ala443Val) proteins were constructed using in silico tools (**Figure 3** and **Supplementary Figure 1**). The comparison between predicted tertiary structures of wt and mutant proteins was performed. p.Asn798Lys, a neutral Asn replaced by the positively charged Lys, formed new interactions between the mutated residue Lys and surrounding residues, including covalent bond with 797Val, hydrogen bonds with 797Val, 820Gly, and 821Phe, and salt bridge with Asp796. p.Arg769Trp, a positively charged Arg mutated to the neutral aromatic residue Trp, made the non-covalent interaction between Arg769 and Tyr772 disappear, resulting in new interactions between the mutated residue Trp769 and surrounding residues Ala856 and Ala855. A strong interaction *via* the hydrogen bond network was observed between Asn592 and five surrounding residues (i.e., Glu596, Asp630, Arg595, Asp630, and Leu603). However, the replacement of Ser592 broke the hydrogen bond interactions with Arg595, Asp630, and Leu603. For p.Ala443Val, non-covalent interactions were observed between Ala443 and Val440, Gln446, Ile447, Phe678, and Leu686. When Ala443 was replaced by Val, the hydrogen bond interactions with Gln446 and Ile447 were disappeared.

### *In Vitro* Functional Characterization of Mutant Proteins

**Figure 4:**
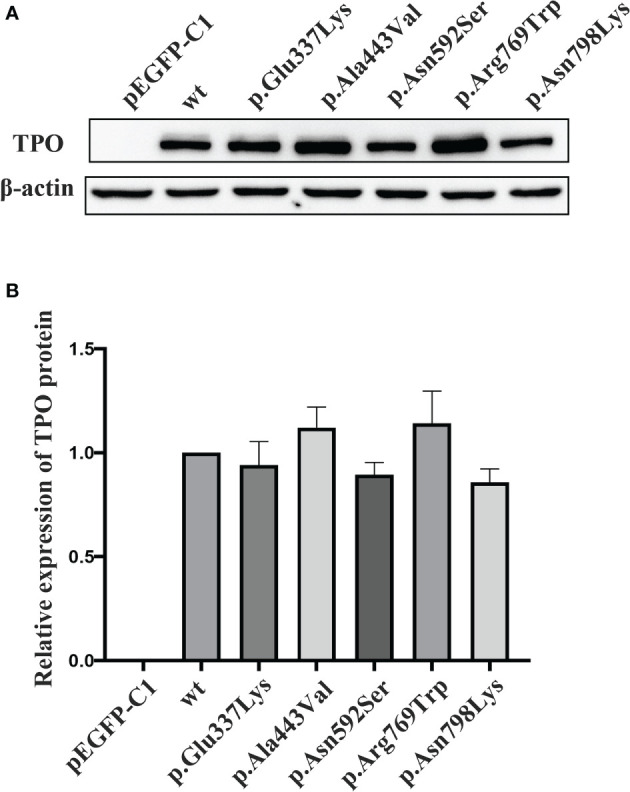
Protein expression level of wild-type and mutant TPOs. **(A)** Western blotting results of wild-type and mutant TPO proteins. **(B)** Relative expression level of tested proteins in relation to wild-type TPO protein. wt, wild-type.

*In vitro* function studies were performed on 4 variants (i.e., p.Asn798Lys, p.Arg769Trp, p.Asn592Ser, and p.Ala443Val) carried by two compound heterozygotes (E160098 and E170009) and 1 documented pathogenic variant p.Glu337Lys. Immunoblotting experiment showed that the relative expression level of these five mutant proteins were comparable with that of the wt TPO protein (p > 0.05, **Figure 4**).

**Figure 5:**
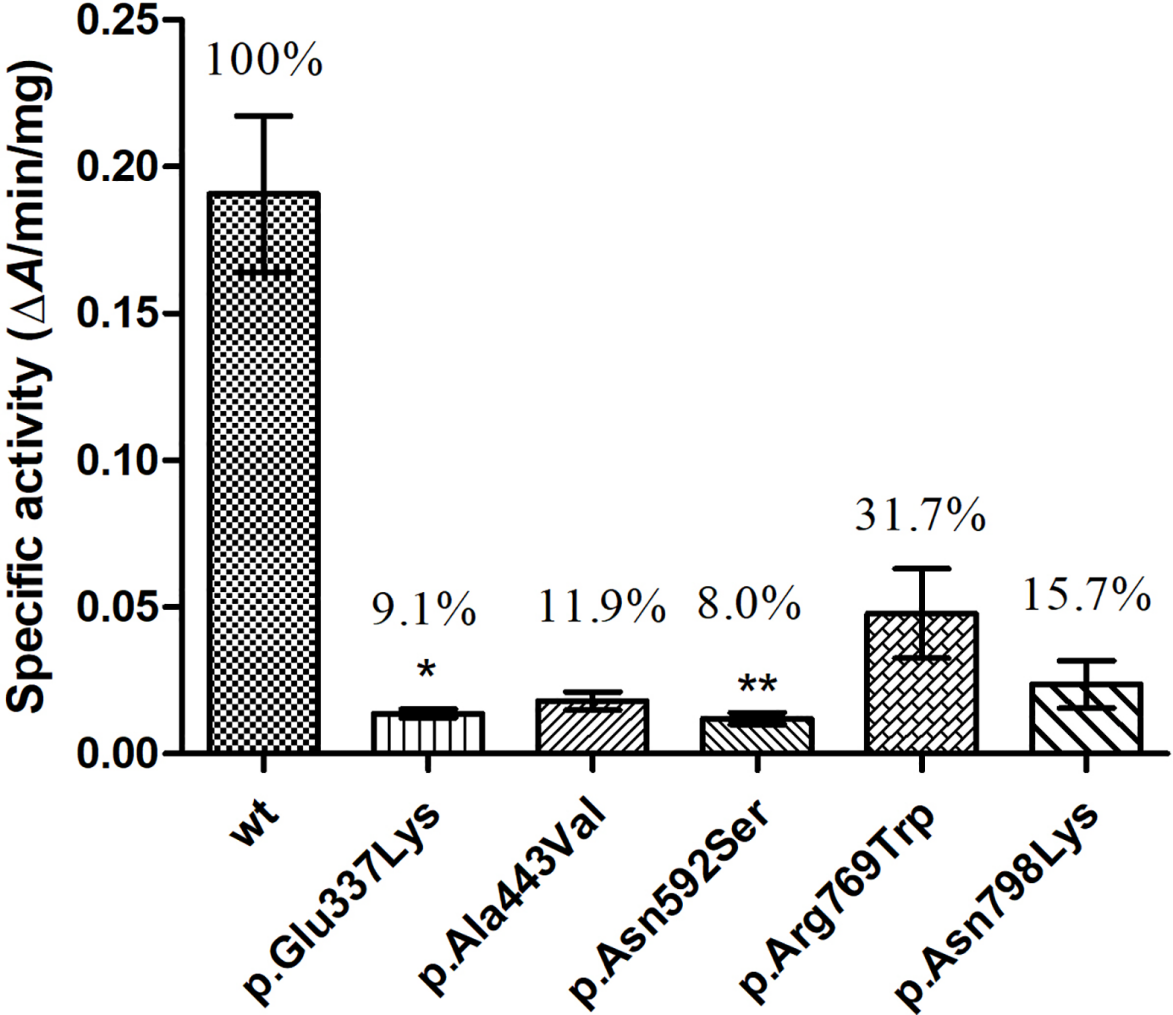
Enzymatic activity of wild-type (wt) and mutant TPO proteins. *P < 0.05; **P < 0.01. P value refers to Dunnett’s test, all compared with wild-type TPO protein.

Guaiacol was used as substrate, and the specific enzymatic activities of wt and mutant TPO proteins were determined. The specific activity of these five mutants were reduced to about 8–32% of that of wt protein, and the activities of p.Glu337Lys (p < 0.05) and p.Ala443Val (p < 0.05) were significantly decreased compared with that of the wt protein (**Figure 5**).

## Discussion

In this study, we screened and identified *TPO* gene mutations in 219 patients with CH from northwest China, which had a high incidence of CH. The mutation spectrum of the *TPO* gene was analyzed, and the relationship between the *TPO* genotype and phenotype in patients with CH were explored to elucidate the pathogenic mechanism of *TPO* mutations in CH occurrence and provide a strong basis for the diagnosis, prognosis, and treatment of patients with CH.

First, the mutation spectrum of the *TPO* gene in patients with CH was analyzed. Nineteen rare variants were identified in 17 patients with a detection rate of 7.8%. This value was higher than the 1% *TPO* mutation rate reported in Guangxi located in the south of China (20) and lower than the rate of 10% in Shanghai located in the east of China (21) (**Supplementary Table 3**). Fifteen patients carried one heterozygous mutation, and two patients carried multiple *TPO* mutations. Based on the autosomal recessive pattern of the *TPO* mutation, data suggested that the *TPO* mutation might not be the major genetic factor causing CH in the Chinese population. In addition, c.2268dupT (p.E757fs or p.E757*), a non-sense mutation in the exon 13 of the *TPO* gene, is reported as a hotspot mutation in Taiwanese and Malaysian–Chinese patients and the most common genetic cause of dyshormonogenetic CH with evidence of cofounder effect (10, 30, 32). However, c.2268dupT was only found heterozygous in one patient of the present study, and all other identified variants demonstrated low frequency. These data validated an evident regional and ethnic difference in the role of *TPO* mutation in the pathogenesis of CH.

Second, the relationship between the *TPO* genotype and phenotype of patients with CH in the studied cohort was studied. Previous studies showed that patients with CH and *TPO* mutations often presented with permanent CH and goiter (9, 11, 15). In our study, the two patients carrying multiple *TPO* mutations had severe CH and FT4 levels below 5 pmol/L, indicating that *TPO* biallelic mutations often led to severe CH phenotype. However, this study also confirmed that the *TPO* genotype was not directly related to the patient phenotype. The phenotype of patients carrying a single heterozygous variant remarkably varied, demonstrating light, moderate, and severe CH. Even with the same mutation, the patient’s phenotype was not identical. The thyroid morphology of patients was also varied. Some had goiter, some had normal or no thyroid, indicating that other undetected gene mutations or other factors participated in the final embodiment of the phenotype.

Third, the effects of identified variants on the TPO protein structure and function were investigated. Consistent with those in previous studies (9, 15, 16, 20, 33, 34), the majority of detected variants were located in the MPO-like domain of the TPO protein, which hamper normal biological functions. In patients with *TPO* mutations, two cases harbored multiple mutations, i.e., compounds for (1) IVS7-1G>A, p.Ala443Val, and p.Arg769Trp and (2) p.Asn592Ser and p.Asn798Lys. The variants they carried were of either paternal or maternal origin, and none came from one single parent. IVS7-1G>A is a splicing variant affecting the splicing of precursor mRNA, which may lead to the production of inactive protein. The functions of the four missense mutations (p.Ala443Val, p.Arg769Trp, p.Asn592Ser, and p.Asn798Lys) they carried have not been studied before. Thus, *in silico* methods and *in vitro* experiments were performed on these four variants and one previously documented pathogenic variant p.Glu337Lys. Experimental data showed that the protein expression levels of these five mutants were comparable with that of the wt TPO protein, but the enzymatic activities of these mutants were remarkably reduced. p.Asn798Lys, p.Asn592Ser, p.Ala443Val, and p.Glu337Lys only retained less than 20% activity toward guaiacol oxidation. Together with another *TPO* mutation, p.Glu337Lys was previously reported in a patient with a PIOD and goiter and showed residual activity towards guaiacol oxidation (11). In the present study, p.Glu337Lys was found in a patient with goiter, and the experimental data reconfirmed its causal role. Ala443 and Asn592 were all located in the MPO-like domain of TPO protein and highly conserved in the TPO family and in other members of the peroxidase superfamily (**Supplementary Figure 2**). *In silico* programs predicted that p.Ala443Val and p.Asn592Ser might have a deleterious effect on the TPO protein, and the molecular modeling analysis showed that the amino acid substitutions impaired multiple hydrogen-bonding interactions in the protein, which might destabilize the structure of protein. The mutant p.Arg769Trp, located in the CCP-like domain, a positively charged arginine is substituted by the neutral Trp, and the mutant p.Asn798Lys, located in the EGF-like domain, the neutral Asn is replaced by the positively charged Lys. The mutant forms of p.Arg769Trp and p.Asn798Lys all formed multiple new interactions with surrounding residues, which might change the protein conformation and catalytic activity. These findings suggested that these five missense variants might affect the protein function through the alteration of protein structure or conformation but not the protein expression. Based on these above data, we can preclude that compound heterozygous mutations identified in *TPO* were highly likely to be causal for CH.

Several limitations should be addressed in this study. First, some patients in this study changed hospitals during treatment. Thus, complete clinical information was impossible to collect, thereby affecting the in-depth analysis of the *TPO* genotype–phenotype relationship. Second, only a minority of CH cases are genetic diseases caused by single genetic defects, and the majority of CH may be caused by multiple genetic and/or environmental factors (2, 35). Samples should be screened for other genes in subsequent studies to gain an improved understanding of the genetic etiology of CH. Finally, biological function investigations were not performed on all detected variants, and we just paid attention to the variants validated through the family pedigree analysis. In the future, further studies with large sample size and pedigree samples are needed to verify the conclusions obtained in this study and gain a thorough understanding on the molecular mechanism of *TPO* mutation involved in the CH pathogenesis.

In summary, this study conducted a comprehensive screening and identification of *TPO* mutations in 219 patients with CH in northwest China and performed preliminary biological functional studies to reveal the pathogenic mechanism of some important variants. We found *TPO* mutations occurred at a low frequency in Chinese patients with CH and might not be the main genetic factor causing CH. Functional studies of the five *TPO* mutations suggested that these amino acid substitution mutations might affect the protein function by changing the protein structure of the protein, which might result in impaired thyroid hormone synthesis.

## Data Availability Statement

The datasets presented in this study can be found in online repositories. The names of the repository/repositories and accession number(s) can be found in the article/**Supplementary Material**.

## Ethics Statement

The studies involving human participants were reviewed and approved by Urumqi Maternal and Child Health Care Hospital. Written informed consent to participate in this study was provided by the participants’ legal guardian/next of kin.

## Author Contributions

HW and GD conceptualized and designed the study and drafted the initial manuscript. XC collected the sample and analyzed the basic clinical data. WW and YS performed the experiments. HS and HW analyzed the data and interpreted the results. All authors contributed to the article and approved the submitted version of the manuscript and agreed to be accountable for all aspects of the work.

## Funding

This work was supported by the Natural Science Foundation of Xinjiang Uygur Autonomous Region (2019D01A16).

## Conflict of Interest

The authors declare that the research was conducted in the absence of any commercial or financial relationships that could be construed as a potential conflict of interest.

## Publisher’s Note

All claims expressed in this article are solely those of the authors and do not necessarily represent those of their affiliated organizations, or those of the publisher, the editors and the reviewers. Any product that may be evaluated in this article, or claim that may be made by its manufacturer, is not guaranteed or endorsed by the publisher.

## Supplementary Material

Supplementary Figure 1 Computer generated models of the wild type and mutant TPO. Ribbon **(A, C, E, G)** and Stick **(B, D, F, H)** presentation of wild-type and mutant TPO proteins [**(A, B)** p.Asn798Arg; **(C, D)** p.Arg769Trp; **(E, F)** p.Asn592Ser; **(G**, **H)** p.Ala443Val]. These four selected residues as well as those that have an interaction with them are shown as sticks. Non-covalent interactions are shown as dashed lines.

Supplementary Figure 2 Comparisons of the amino acid sequences neighboring the mutations among different species, and different members of peroxidase superfamilies.

Supplementary Table 1 Classification and evidence of detected *TPO* variants.

Supplementary Table 2
*In silico* prediction of the function of detected *TPO* variants.

Supplementary Table 3 Comparison of studies that conducted *TPO* screening in patients with CH.
