# Supplementary material for: Screening and Functional Analysis of TPO Gene Mutations in a Cohort of Chinese Patients With Congenital Hypothyroidism
Source: Front Endocrinol (Lausanne). 2021 Dec 21;12:774941. doi: 10.3389/fendo.2021.774941 (PMC8729100; doi:10.3389/fendo.2021.774941)
Supplement: Supplementary Figure 1 — Computer generated models of the wild type and mutant TPO. Ribbon (A, C, E, G) and Stick (B, D, F, H) presentation of wild-type and mutant TPO proteins [(A, B) p.Asn798Arg; (C, D) p.Arg769Trp; (E, F) p.Asn592Ser; (G, H) p.Ala443Val]. These four selected residues as well as those that have an interaction with them are shown as sticks. Non-covalent interactions are shown as dashed lines. [file DataSheet_1.zip › Supplementary Table 1.docx]

Supplementary Table 1 Classification and evidence of detected *TPO* variants.

| Variants | Classification | PVS1 | PS3 | PM1 | PM2 | | PM3 | PM4 | PM5 | PP1 | PP3 | PP5 | BS3 | BP4 |
| --- | --- | --- | --- | --- | --- | --- | --- | --- | --- | --- | --- | --- | --- | --- |
|  |  | Truncation variants | Well-established in vitro or in vivo functional studies supportive of a damaging effect on the gene or gene product | Located in functional domain | gnomAD East Asian | 1000 genomes (CHB) | A pathogenic variant detected in trans | Protein length changing variant | Novel missense change at an amino acid residue where a different missense change determined to be pathogenic has been seen before | Cosegregation with disease in multiple affected family members | In silico prediction | Reputable source recently reports variant as pathogenic | Well-established in vitro or in vivo functional studies show no damaging effect on protein function or splicing | Multiple lines of computational evidence suggest no impact on gene or gene product |
| p.Gly889Arg | VUS |  |  |  | 0.0003492 | 0 | Yes (Ma et al., 2015) |  |  |  |  |  |  |  |
| p.Pro883Ser | LP |  | Yes (Yoshizawa-  Ogasawara et al., 2016) |  | 0.005409 | 0.0146 |  |  |  |  |  | Yes | B (Yoshizawa-Ogasawara, et al., 2016) | T |
| p.Ser853Leu | LP |  |  | Transmembrane domain | 0.0001157 | / | / |  |  |  |  |  |  |  |
| p.Arg846Trp | VUS |  |  |  | 0.00159 | 0.0049 |  |  |  |  |  |  |  |  |
| p.Asn798Lys | LP |  |  | EGF-like domain | / | / | Yes，this study |  |  |  | D |  |  |  |
| p.Arg769Trp | LP |  |  | CCP-like domian | 0.003235 | 0 | Yes，this study |  |  |  |  |  |  |  |
| p.Glu757* | P | Yes | Yes(Lee et al., 2014) | CCP-like domian | 0.00159 | / | Yes (Lee et al., 2014; Ma, et al., 2012) | Yes |  | Yes |  | Yes |  |  |
| p.Asn674Ser | VUS |  |  | An peroxidase | 0 | / |  |  |  |  |  |  |  |  |
| p.Gly673Lys | VUS |  |  | An peroxidase | / | 0.0049 |  |  |  |  | D |  |  |  |
| p.Gly650Glu | VUS |  |  | An peroxidase | 0.0001156 | 0.0049 |  |  |  |  | D |  |  |  |
| p.Asn592Ser | LP |  |  | An peroxidase | / | / | Yes，this study |  |  |  |  |  |  |  |
| p.Ser571Arg | VUS |  |  | An peroxidase | / | / |  |  |  |  |  |  |  |  |
| p.Ala443Val | LP |  |  | An peroxidase | / | / | Yes，this study |  | Yes |  | D |  |  |  |
| p.Arg361Leu | LP |  | Yes (Yoshizawa-Ogasawara, et al., 2016) | An peroxidase | 0.009273 | 0.0194 |  |  |  |  |  | Yes |  |  |
| p.Glu337Lys | P |  | Yes (Zhang, et al., 2020) | An peroxidase | / | / | Yes (Zhang, et al., 2020) |  |  |  |  | Yes |  |  |
| p.Ser309Pro | VUS |  |  | An peroxidase | / | / |  |  |  |  |  |  |  |  |
| p.Arg279Trp | VUS |  |  | An peroxidase | / | / |  |  |  |  |  |  |  |  |
| IVS7-1G>A | P | Yes |  |  | 0.00005722 | / | Yes，this study |  |  |  | D |  |  |  |
| p.Pro135His | VUS |  |  |  | / | / |  |  |  |  |  |  |  |  |

P, pathogenic; LP, likely pathogenic; VUS, variants of uncertain significance; LB, likely benign; D, damaged; B, benign; CHB, Han Chinese in Beijing, China; PVS1, null variant (nonsense, frameshift, canonical ±1 or 2 splice sites, initiation codon, single or multi-exons deletion) in a gene where LOF is a known mechanism of disease; PS3, well-established in vitro or in vivo functional studies supportive of a damaging effect on the gene or gene product; PM1, located in a mutational hot spot and/or critical and well-established functional domain; PM2, Absent from controls (or at extremely low frequency if recessive) in public population databases; PM3, for recessive disorders, detected in trans with a pathogenic variant; PM4, protein length changes as a result of in-frame deletions/insertions in a non-repeat region, or stop-loss variants; PM5, Novel missense change at an amino acid residue where a different missense change determined to be pathogenic has been seen before; PP1, Cosegregation with disease in multiple affected family members in a gene definitively known to cause the disease; PP3, multiple lines of computational evidence support a deleterious effect on the gene or gene product (detailed prediction results shown in Supplemental Table 1); PP5, Reputable source recently reports variant as pathogenic, but the evidence is not available to the laboratory to perform an independent evaluation; BS3, Well-established *in vitro* or *in vivo* functional studies show no damaging effect on protein function or splicing; BP4, Multiple lines of computational evidence suggest no impact on gene or gene product (including conservation, evolutionary and splicing impact).
