# Supplementary material for: Screening and Functional Analysis of TPO Gene Mutations in a Cohort of Chinese Patients With Congenital Hypothyroidism
Source: Front Endocrinol (Lausanne). 2021 Dec 21;12:774941. doi: 10.3389/fendo.2021.774941 (PMC8729100; doi:10.3389/fendo.2021.774941)
Supplement: Supplementary Figure 1 — Computer generated models of the wild type and mutant TPO. Ribbon (A, C, E, G) and Stick (B, D, F, H) presentation of wild-type and mutant TPO proteins [(A, B) p.Asn798Arg; (C, D) p.Arg769Trp; (E, F) p.Asn592Ser; (G, H) p.Ala443Val]. These four selected residues as well as those that have an interaction with them are shown as sticks. Non-covalent interactions are shown as dashed lines. [file DataSheet_1.zip › Supplementary Table 2.docx]

Supplementary Table 2 In silico prediction of the function of detected *TPO* variants.

| Variants | SIFT | PolyPhen-2 | Mutation Taster | M-CAP | Result |
| --- | --- | --- | --- | --- | --- |
| p.Gly889Arg | Damaging | Possibly_damaging | Polymorphism | tolerable |  |
| p.Pro883Ser | Tolerable | Benign | polymorphism | Likely Benign | T |
| p.Ser853Leu | tolerable | benigh | Polymorphism | damaging |  |
| p.Arg846Trp | Damaging | Probably_damaging | polymorphism | Damaging |  |
| p.Asn798Lys | Damaging | Probably_damaging | Disease_causing | Damaging | D |
| p.Arg769Trp | tolerable | Possibly_damaging | Polymorphism | NA |  |
| p.Asn674Ser | tolerable | benigh | Polymorphism | damaging |  |
| p.Gly673Lys | Damaging | Possibly_damaging | disease causing | damaging | D |
| p.Gly650Glu | Damaging | Possibly_damaging | disease causing | damaging | D |
| p.Asn592Ser | Damaging | Probably_damaging | Disease_causing | Damaging | D |
| p.Ser571Arg | tolerable | benigh | Polymorphism | damaging |  |
| p.Ala443Val | Damaging | Possibly_damaging | disease causing | damaging | D |
| p.Arg361Leu | Tolerable | Probably_damaging | disease causing | NA |  |
| p.Glu337Lys | Tolerable | Probably_damaging | Polymorphism | Damaging |  |
| p.Ser309Pro | Tolerable | Benign | polymorphism | Damaging |  |
| p.Arg279Trp | Damaging | Probably_damaging | Polymorphism | Damaging |  |
| p.Pro135His | Tolerable | Probably_damaging | Polymorphism | NA |  |

SIFT, Sorting intolerant from tolerant; PolyPhen-2, Polymorphism Phenotyping v2; M-CAP, Mendelian Clinically Applicable Pathogenicity; D, deleterious; T, tolerable.
