# Supplementary material for: Screening and Functional Analysis of TPO Gene Mutations in a Cohort of Chinese Patients With Congenital Hypothyroidism
Source: Front Endocrinol (Lausanne). 2021 Dec 21;12:774941. doi: 10.3389/fendo.2021.774941 (PMC8729100; doi:10.3389/fendo.2021.774941)
Supplement: Supplementary Figure 1 — Computer generated models of the wild type and mutant TPO. Ribbon (A, C, E, G) and Stick (B, D, F, H) presentation of wild-type and mutant TPO proteins [(A, B) p.Asn798Arg; (C, D) p.Arg769Trp; (E, F) p.Asn592Ser; (G, H) p.Ala443Val]. These four selected residues as well as those that have an interaction with them are shown as sticks. Non-covalent interactions are shown as dashed lines. [file DataSheet_1.zip › Supplementary Table 3.docx]

Supplementary Table 3 Comparison of studies that conducted *TPO* screening in patients with CH.

| Study | Present study | Fu,et al.,2016 (ref.20 ) | Zhang,et al.,2020 (ref.21) | NIU,et al.,2002 (ref.10) | Lee,et al.,2011 (ref.22) | Avbelj,et al.,2007 (ref.15) |
| --- | --- | --- | --- | --- | --- | --- |
| Patients | 219 CH patients | 192 CH patients | 230 CH patients | 7 CH patients with total iodide organification defect | 193 CH patients | 43 patients with dyshormonogenetic CH from 39 unrelated families |
| Region | XinJiang, China | Guangxi, China | Shanghai, China | Taiwan, China | Korea | Slovakia |
| TPO mutation detection rate (n) | 7.8% (17) | 1% (2) | 10% (23) | 100% (7) | 3.6% (7) | 46% (20) |
| Number of TPO variants detected | 19 | 3 | 35 | 3 | 7 | 7 |
| Carriers with multiple TPO mutations | 2 | 2 | 13 | 7 | 1 | 7 |
| Mutations found in multiple patients | p.Pro883Ser, p.Arg846Trp | N.D. | p.Glu757*, p.Ala443Pro, p.Cys269Ser, and p.224_224del | p.Glu757* | p.Pro883Ser | A397PfsX76, A477_N483del |
| Number of patients with c.2268dupT  (p.Glu757*) | 1 | N.D. | 9 | 6 | N.D. | N.D. |

N.D. Not detected.
