# Supplementary figures and images for: Screening and Functional Analysis of TPO Gene Mutations in a Cohort of Chinese Patients With Congenital Hypothyroidism
Source: Front Endocrinol (Lausanne). 2021 Dec 21;12:774941. doi: 10.3389/fendo.2021.774941 (PMC8729100; doi:10.3389/fendo.2021.774941)

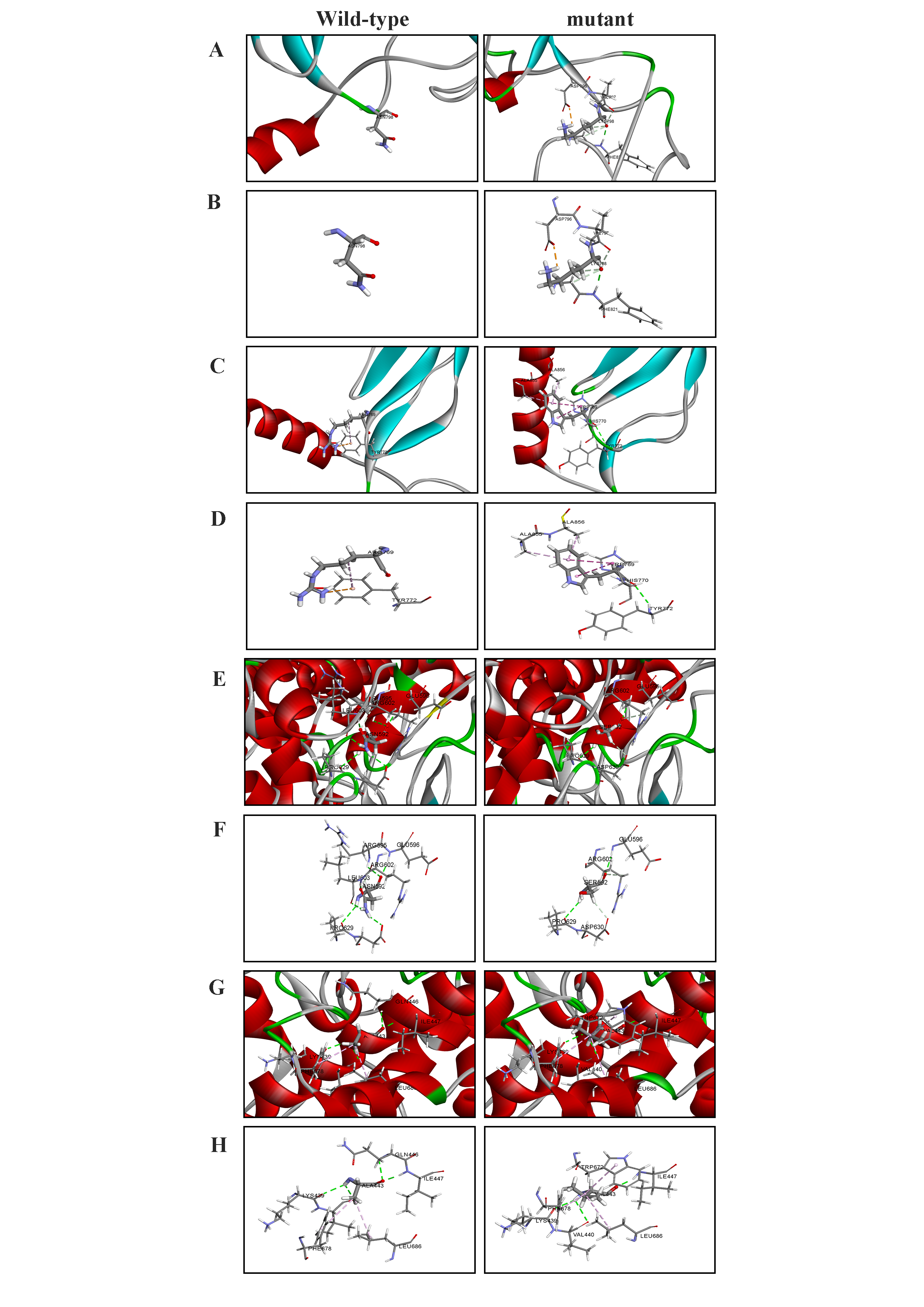

Supplement: Supplementary Figure 1 — Computer generated models of the wild type and mutant TPO. Ribbon (A, C, E, G) and Stick (B, D, F, H) presentation of wild-type and mutant TPO proteins [(A, B) p.Asn798Arg; (C, D) p.Arg769Trp; (E, F) p.Asn592Ser; (G, H) p.Ala443Val]. These four selected residues as well as those that have an interaction with them are shown as sticks. Non-covalent interactions are shown as dashed lines. [file DataSheet_1.zip › Supplementary Figure 1.tif]

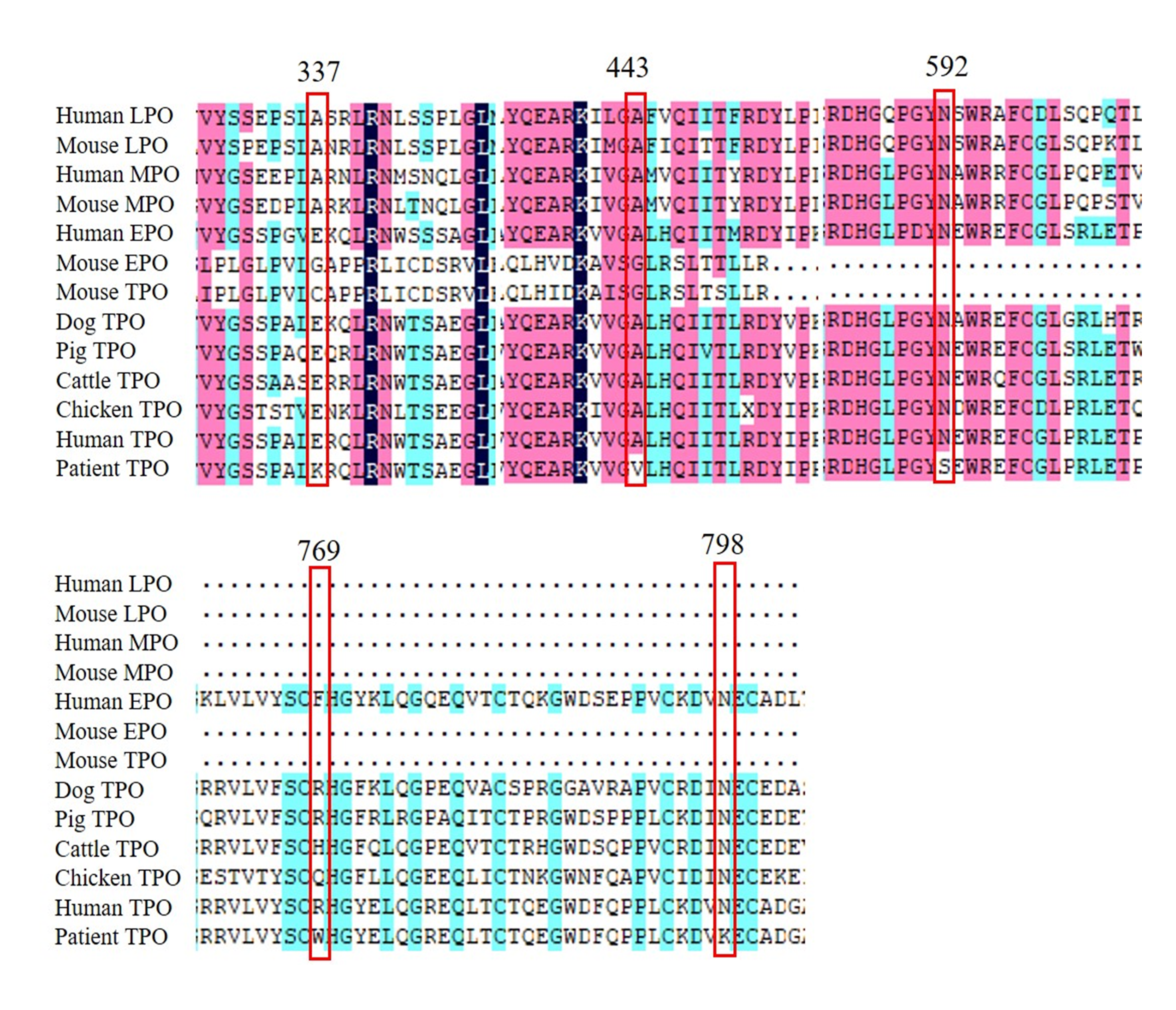

Supplement: Supplementary Figure 1 — Computer generated models of the wild type and mutant TPO. Ribbon (A, C, E, G) and Stick (B, D, F, H) presentation of wild-type and mutant TPO proteins [(A, B) p.Asn798Arg; (C, D) p.Arg769Trp; (E, F) p.Asn592Ser; (G, H) p.Ala443Val]. These four selected residues as well as those that have an interaction with them are shown as sticks. Non-covalent interactions are shown as dashed lines. [file DataSheet_1.zip › Supplementary Figure 2.tif]
